# De novo SIX2 activation in human kidneys treated with neonatal kidney stem/progenitor cells

**DOI:** 10.1111/ajt.17164

**Published:** 2022-08-11

**Authors:** Fanny Oliveira Arcolino, Sarah Hosgood, Sara Akalay, Nina Jordan, Jean Herman, Tegwen Elliott, Koenraad Veys, Kurt Vermeire, Ben Sprangers, Michael Nicholson, Lambertus van den Heuvel, Elena Levtchenko

**Affiliations:** ^1^ Department of Development and Regeneration Cluster Woman and Child, Laboratory of Paediatric Nephrology, KU Leuven Leuven Belgium; ^2^ Department of Surgery University of Cambridge, Addenbrookes Hospital Cambridge UK; ^3^ Department of Microbiology, Immunology and Transplantation Laboratory of Molecular Immunology, Rega Institute,KU Leuven Leuven Belgium; ^4^ Interface Valorisation Platform (IVAP) KU Leuven Leuven Belgium; ^5^ Department of Paediatric Nephrology and Solid Organ Transplantation University Hospitals Leuven Leuven Belgium; ^6^ Department of Paediatric Nephrology University Hospitals Leuven UZ Leuven Leuven Belgium; ^7^ Department of Microbiology, Immunology and Transplantation Rega Institute, Laboratory of Virology and Chemotherapy KU Leuven Leuven Belgium; ^8^ Department of Internal Medicine, Division of Nephrology University Hospitals Leuven, UZ Leuven Leuven Belgium; ^9^ Department of Paediatric Nephrology Radboud University Medical Center Nijmegen Netherlands

**Keywords:** basic (laboratory) research/science, immunosuppression/immune modulation, kidney transplantation/nephrology, regenerative medicine, stem cells, organ perfusion and preservation, tissue injury and repair

## Abstract

During development, nephron structures are derived from a SIX2+ stem cell population. After 36 weeks of gestation, these cells are exhausted, and no new nephrons are formed. We have previously described a non‐invasive strategy to isolate and expand the native SIX2+ kidney stem cells from the urine of preterm neonates, named neonatal kidney stem/progenitor cells (nKSPC). Here, we investigated the safety and feasibility of administering nKSPC into human kidneys discarded for transplantation during normothermic machine perfusion (NMP) and evaluated the regenerative and immunomodulatory potential of nKSPC treatment. We found that nKSPC administration during NMP is safe and feasible. Interestingly, nKSPC induced the de novo expression of SIX2 in proximal tubular cells of the donor kidneys and upregulated regenerative markers such as *SOX9* and *VEGF*. This is the first time that SIX2 re‐expression is observed in adult human kidneys. Moreover, nKSPC administration significantly lowered levels of kidney injury biomarkers and reduced inflammatory cytokine levels via the tryptophan‐IDO‐kynurenine pathway. In conclusion, nKSPC is a novel cell type to be applied in kidney‐targeted cell therapy, with the potential to induce an endogenous regenerative process and immunomodulation.

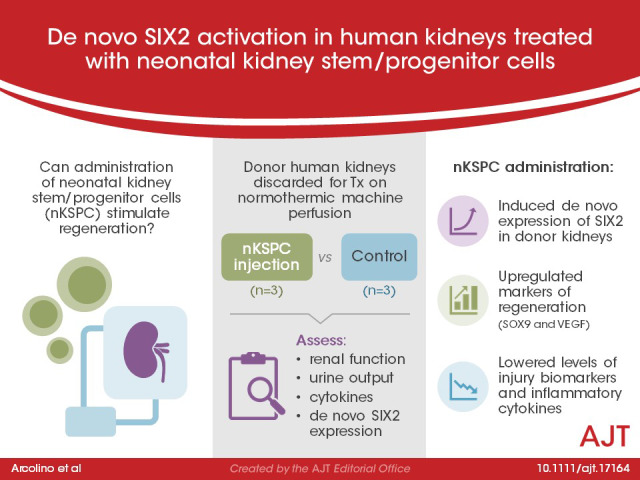

## Graphical abstract

In human discarded kidneys, administration of neonatal kidney stem/progenitor cells during normothermic machine perfusion exerts immunomodulatory effects and induces de novo expression of a developmental transcription factor and markers of regeneration in proximal tubular cells.

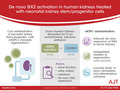

**Abbreviations**
AKI: acute kidney injury
CKD: chronic kidney disease
DCD: donation after circulatory death
ESKD: end‐stage kidney disease
HIF: hypoxia‐inducible factors
HPLC‐MS: High‐performance liquid chromatography—mass spectrometry
IDO: indoleamine 2,3‐dioxygenase
IFN‐γ: interferon gamma
IL: interleukin
IRI: ischemia‐reperfusion injury
KIM1: kidney injury molecule 1
LTL: Lotus Tetragonolobus (Asparagus Pea) Lectin
MLR: mixed lymphocyte reaction
MSC: mesenchymal stem cells
NGAL: neutrophil gelatinase‐associated lipocalin
nKSPC: neonatal kidney stem/progenitor cells
NMP: normothermic machine perfusion
PBMC: peripheral blood mononuclear cells
PGE2: prostaglandin E2
qPCR: quantitative polymerase chain reaction
RBF: renal blood flow
TNF‐α: tumor necrosis factor‐α
VEGF: vascular endothelial growth factor

## 1 INTRODUCTION

Chronic kidney disease (CKD) is caused by the irreversible loss of nephrons, the structural, and functional units of the kidney. Global mortality from CKD is estimated at above 1 million people per year.^1^ Kidney transplantation is the preferred treatment for patients with end‐stage kidney disease (ESKD) and it is anticipated that by 2030, 5.4 million people worldwide will necessitate a kidney graft.^1^ Due to the shortage of donor organs, only 25% of patients will receive a transplant. To increase the number of kidneys for transplantation, inferior‐quality grafts, such as those from donation after circulatory death (DCD) are now considered,^2^ which urges for strategies to improve the quality of these kidneys, such as ex vivo normothermic machine perfusion (NMP).^3^, ^4^

NMP of kidneys from marginal donors offers the opportunity to deliver therapies directly to the organ excluding systemic effects and yielding an ideal pre‐clinical platform for investigating kidney‐targeted cell therapy.

We have previously established a non‐invasive strategy to isolate and expand neonatal kidney stem/progenitor cells (nKSPC) from the urine of preterm neonates.^5^ nKSPC represent a unique kidney stem/progenitor cell type derived from developing kidneys, the only period in which authentic stem cells are present in the human kidney.^6^, ^7^, ^8^ nKSPC have endogenous *SIX2* expression, a transcription factor active in kidney cap mesenchyme cells, which is responsible for cells self‐renewal and survival, and its cessation drives differentiation of cells to form nephrons.^9^

This SIX2+ cells population does not persist in the postnatal kidney, which limits the regenerative potential of the organ upon injury.^7^ Still, substantial repair of tubular cells in response to acute kidney injury (AKI), elicits an ongoing discussion about the source of regeneration. Continuous and established AKI trigger maladaptive repair responses, putting patients at high risk of kidney failure, while no therapy exists to promote effective endogenous regeneration, making this, a high priority goal in nephrology.^10^

In this study, we administered nKSPC to donor human kidneys during NMP. We show for the first time that treatment with nKSPC induces the de novo expression of SIX2 in proliferating proximal tubule epithelial cells (PTEC) of donor kidneys, suggesting initiation of an endogenous regenerative process. This phenomenon is confirmed in in vitro co‐cultures of injured PTEC with nKSPC. Moreover, nKSPC treatment modulates the immune response by reducing pro‐inflammatory cytokines. This immunomodulatory effect relies on the mechanism of activation of the tryptophan‐IDO‐kynurenine pathway, as shown in vitro and confirmed in perfusate samples.

## 2 MATERIAL AND METHODS

### 2.1 Ethical statement

The use of nKSPC was approved by the ethical board of KU Leuven (s53345) and an informed consent form was signed by the parents or legal guardian. Peripheral blood mononuclear cells (PBMCs) were isolated from blood samples of healthy volunteers at the Red Cross of Mechelen, Belgium, under an informed consent form signed by each donor. In the UK ethical approval was granted by the National Research Ethics Committee (NRES) to use the discarded human kidneys for research (15/NE/0408). Consent for research was obtained by Specialist Nurses in Organ Donation (SNODs) from the donor families.

### 2.2 Normothermic machine perfusion (NMP)

nKSPC^5^ were labeled with 3 μg/ml of the red fluorescent dye CellTracker CM‐DiI (Invitrogen) the day before injection into the human kidneys (Figure S1).

1 × 10^7^ nKSPC were added to 50 ml Ringer's solution and flushed into the renal artery (5–10 min) before NMP. NMP was carried out by perfusing the kidneys with an oxygenated red cell‐based solution at 36°C and a mean arterial pressure of 75 mm Hg using adapted pediatric cardiopulmonary bypass technology as previously described.^4^

### 2.3 Quantitative polymerase chain reaction (qPCR)

mRNA from cells was isolated using RNeasy Mini or Micro Kit (Qiagen GmbH, Hilden, Germany), according to the manufacturer's protocol. RNA from tissue was extracted using TriPure Isolation Reagent (Sigma–Aldrich). RNA was synthesized to cDNA using a mix of Oligo (dT) 12–18 Primer, random primers, dNTP mix (100 mM), and SuperScript™ III Reverse Transcriptase, all from Invitrogen. qPCR was performed on a CFX96™ Real‐Time PCR Detection System, using Platinum™ SYBR™ Green qPCR SuperMix‐UDG w/ROX (Thermo Fisher), 10 μM of primers, and 1 μl of cDNA (5 ng/μl). qPCR data were retrieved and processed using the CFX Manager™ software (Bio‐Rad, USA). All primers used are specified in Table S1.

### 2.4 Cytokines and NGAL measurements

IL‐6 (R&D Systems) and IL‐10 (R&D Systems) were measured using ELISA following the manufacturer's instructions. IL‐10 assay was carried out on undiluted perfusate. IL‐6 samples were optimized to fit within the assay standard curve.

Human neutrophil gelatinase‐associated lipocalin (NGAL) ELISA assay (Abcam) was carried out following the manufacturer's instructions. Urine was assayed at 100× dilution.

### 2.5 Immunofluorescence staining

5 μm thickness sections were fixed with 4% formaldehyde and quenched with 0.5% Triton X‐100 (Sigma‐Aldrich). For blocking, 1% BSA (Sigma‐Aldrich) and 0.3% Triton X‐100 (Sigma‐Aldrich) in PBS were used. Specification of antibodies is described in Table S2 and validation of the SIX2 antibodies is presented in Figure S1.

Microscopy was performed on a Nikon Eclipse Ci microscope (Tokyo, Japan) or the Zeiss® LSM 880—Airyscan (Carl Zeiss Microscopy GmbH, Germany) supported by Hercules AKUL/15/37_GOH1816N and FWO G.0929.15 to Pieter Vanden Berghe, KU Leuven. Images were processed and analyzed using Zeiss ZEN Black and Blue imaging software and Fiji/ImageJ.

### 2.6 In vitro co‐cultures

Thirty thousand conditionally immortalized proximal tubule epithelial cells (ciPTEC) were injured with 20 ng/ml of tumor necrosis factor‐α (TNF‐α) and 100 ng/mL of interferon‐gamma (IFN‐γ)^11^ in hypoxia (1% O_2_)^12^ to mimic the ischemic environment of donor kidneys or in control conditions for 24 h. Then, the medium was removed and ciPTEC was incubated with 30 000 CM‐DiI labeled nKSPC or bone marrow‐derived mesenchymal stem cells (MSC) for 24. After incubation, cells were fixed and stained for SIX2 following the protocol in 2.5.

### 2.7 Human mixed lymphocyte reaction (MLR)

Responder cells were 100 000 human freshly obtained PBMCs. Stimulators were 40 000 RPMI 1788 cells (ATCC, USA). RPMI 1788 cells and nKSPC were growth‐inhibited with 96 nM mitomycin C (Kyowa®, Takeda Belgium) for 20 min at 37°C.

DNA synthesis of the responder cells was assayed by the addition of 10 *μ*Ci ^3^H‐thymidine (Perkin Elmer, Zaventem, Belgium) during the last 18 h of culture. Radioactivity was counted in a scintillation counter (TopCount, Perkin Elmer, Zaventem, Belgium). As an internal control, in all experiments, we also used bone marrow‐derived mesenchymal stem cells (MSC) as responder cells for comparison with the nKSPC effect (Figure S2).

Inhibition of indoleamine 2,3‐dioxygenase (IDO) was evaluated after 3 days of MLR + nKSPC co‐culture using 0.1 mM 1‐methyl tryptophan (1‐MT).^13^

### 2.8 Analysis of cytokines by U‐plex

The supernatant of the MLR co‐cultures was used for quantification of IFN‐γ, TNF‐α, IL‐1β, IL‐6, IL‐8, IL‐10, IL‐12 (p70), IL‐13, IL‐17A, IL‐33 using a MSD U‐plex platform (Mesoscale Discovery, Rockville, USA), according to the manufacturer's instructions.

### 2.9 In vitro quantitative assays

Human IDO and prostaglandin E2 (PGE2) were measured in the supernatants of MLR co‐cultures using the IDO DuoSet® Elisa (DY6030‐05) and Prostaglandin E2 Parameter Assay Kit (KGE004B) from R&D Systems, Minneapolis, USA, according to manufacturer's instructions.

### 2.10 Tryptophan and kynurenine measurements by HPLC‐MS

Polar metabolites were extracted using 100% methanol. 50 μl of plasma were added to 545 μl of methanol 100% and 5 ul of Deuterated Internal Standard Mix (1.5 μM of L‐Kynurenine‐d4 and 40 μM of L‐Tryptophan‐indole d5). The supernatant was resuspended in 50 μl of Formic Acid 0.1% and transferred to appropriate mass spectrometer vials.

Fifteen microliters of the sample were injected into a HILIC column (Poroshell 120 HILIC‐Z PEEK, Agilent).

Data collection was performed using the Xcalibur software (Thermo Scientific). The data analyses were performed by integrating the peak areas (El‐Maven—Polly—Elucidata). Absolute concentrations were calculated based on the abundances of the labeled and non‐labeled compounds.

### 2.11 Statistics

All data were analyzed in GraphPad Prism 9.1.0 (221) (GraphPad Software, La Jolla California USA, www. Graphpad.com). qPCR data were analyzed using multiple unpaired t tests comparing NMP group (*n* = 3) versus NMP + nKSPC (*n* = 3). MLRs were analyzed by one‐way ANOVA. Details of analysis for each experiment are described in the legend of the corresponding figures.

## 3 RESULTS

### 3.1 nKSPC administration into human donor kidneys during NMP is safe and feasible

In order to evaluate the safety and feasibility of the administration of nKSPC into donor kidneys during NMP, we perfused six human kidneys (*n* = 6), of which 3 controls (only NMP), and 3 experimental (NMP + nKSPC) (Table 1). These DCD kidneys were subjected to at least 17 h of cold ischemia time (CIT). Donors' ages ranged from 39 to 67 years and the kidneys were declined for transplantation due to damaged vessels or poor perfusion at the time of retrieval.

**TABLE 1:** Demographic characteristics of discarded kidneys perfused with or without nKSPC

	NMP #1 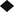	NMP #2 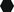	NMP #3 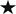	NMP + nKSPC #1 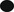	NMP + nKSPC #2 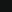	NMP + nKSPC #3 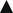
Donor age (y)	53	59	67	54	59	39
Gender	Male	Female	Female	Male	Male	Male
Cause of death	ICH	ICH	ICH	ICH	Hypoxic brain injury	Hypoxic brain injury
Reason for decline	Damaged ureter	Damaged artery	Damaged artery	Diseased artery	Poor perfusion	Poor perfusion
Terminal Cr (μmol/L)	106	65	64	76	70	96
Kidney	Left	Right	Right	Left	Left	Right
WIT (min)	17	18	9	12	12	11
CIT (min)	1674	1200	1647	1441	1377	1054

*Note*: The symbols are used to represent the individual kidneys in graphical analysis.

Abbreviations: CIT, cold ischemia time; Cr, creatinine; ICH, intracranial hemorrhage; NMP + nKSPC, normothermic machine perfusion with neonatal kidney stem/progenitor cells; NMP, normothermic machine perfusion; WIT, warm ischemia time; y, years.

Pre‐labeled nKSPC were flushed into the renal artery (5–10 min) before NMP. Tissue samples were collected before nKSPC infusion (0 h = pre); 5 min after nKSPC infusion (flush); 2 h of NMP (2 h); 4 h of NMP (4 h) and 6 h of NMP (6 h) (Table 2). To assess physiological parameters in real‐time, perfusate samples were collected pre NMP, then perfusate and urine samples, every hour of NMP.

**TABLE 2:** Experimental conditions of biopsies collected from the six human kidneys perfused with normothermic machine perfusion (NMP), with or without the addition of neonatal kidney stem/progenitor cells (nKSPC)

Biopsy time‐point	Condition	Normothermic machine perfusion		nKSPC administration	
		Control group (NMP only)	nKSPC group (NMP + nKSPC)	Control group (NMP only)	nKSPC group (NMP + nKSPC)
Pre	Pre‐perfusion; kidneys stored on ice	✕	✕	✕	✕
Flush	nKSPC injected into the renal artery	✕	✕	✕	✓
2 h cortex	Perfusion for 2 h	✓	✓	✕	✓
4 h cortex	Perfusion for 4 h	✓	✓	✕	✓
6 h cortex	Perfusion for 6 h	✓	✓	✕	✓
6 h medulla	Perfusion for 6 h	✓	✓	✕	✓

**FIGURE 1:**
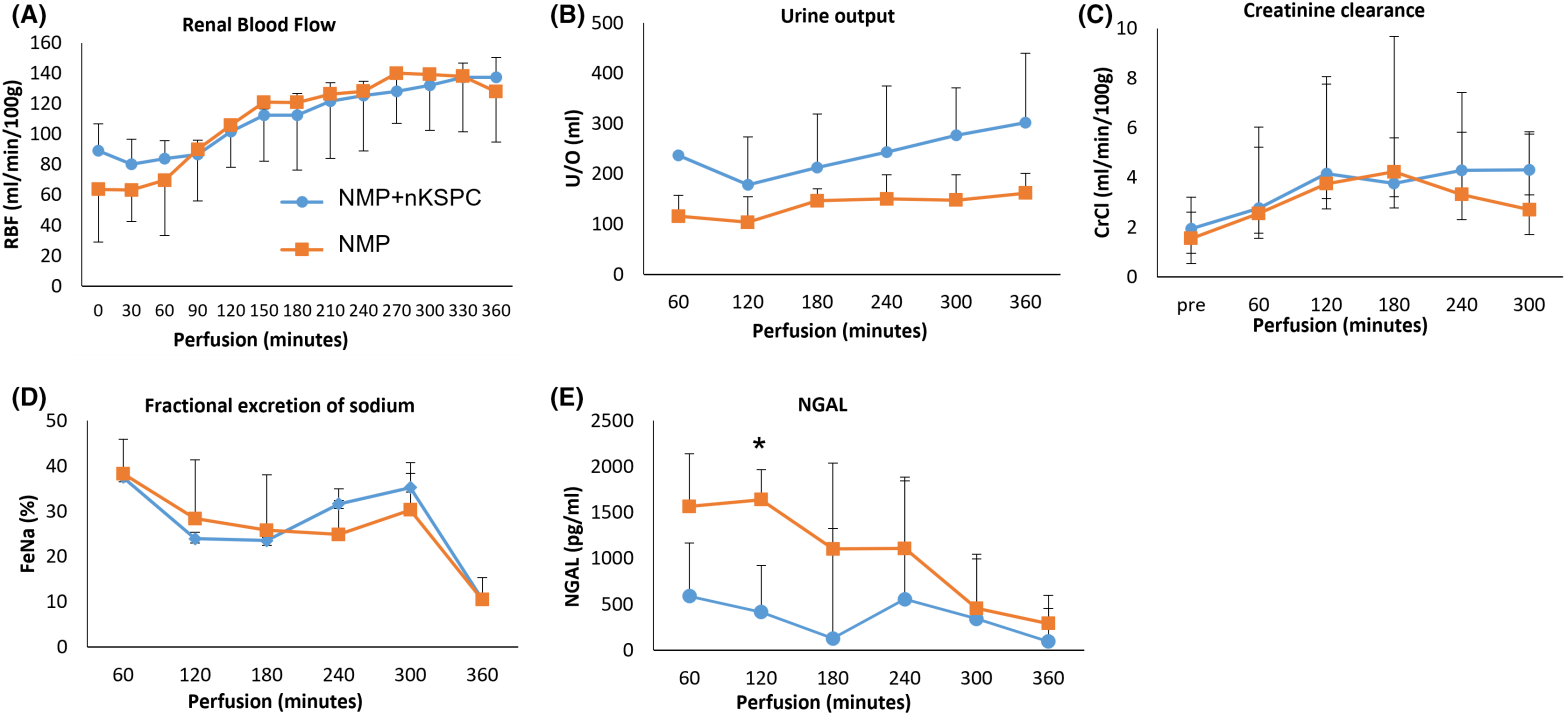
nKSPC administration maintained the function of discarded human kidneys perfused ex‐vivo in normothermic machine perfusion (NMP). (A) Kidneys were injected with nKSPC 5–10 min prior to NMP (*n* = 3) and compared with a control group (*n* = 3). The mean renal blood flow (RBF) during 6 h of NMP. (B) The mean urine output during 6 h NMP. (C) Mean levels of creatinine clearance during 6 h NMP. (D) Mean levels of fractional excretion of sodium during 6 h of NMP. (E) Mean levels of neutrophil gelatinase‐associated lipocalin (NGAL) during 6 h of NMP. Error bars represent the mean with standard deviations.

The administration of nKSPC had no adverse effects on perfusion parameters or renal function during NMP. All kidneys produced urine and appeared evenly perfused. The renal blood flow (RBF) increased during perfusion in both the nKSPC‐treated and control groups. There was no statistically significant difference in RBF levels between the groups (mean; nKSPC 111.4 ± 6.0 vs. control 110.2 ± 33.5 ml/min/100 g 𝑝 = .959; Figure 1A). The nKSPC‐treated kidneys produced a higher volume of urine, but this did not reach statistical significance (mean total urine output; nKSPC 1448 ± 105 vs. control 824 ± 245 ml; 𝑝 = .219; Figure 1B). Other measures of renal and tubular cell function, such as creatinine clearance and fractional excretion of sodium, were also similar between the two groups (mean creatinine clearance; nKSPC 3.5 ± 1.9 vs. control 3.0 ± 3.7 ml/min/100 g 𝑝 = .848; Figure 1C), (mean fractional excretion of sodium; nKSPC 27.1 ± 1.0 vs. control 26.4 ± 9.4% 𝑝 = .909; Figure 1D).

Urinary levels of early kidney injury biomarker NGAL were significantly lower in NMP + nKSPC kidneys (𝑝 = .032). (Figure 1E).

### 3.2 nKSPC can be traced within the perfused kidneys

**FIGURE 2:**
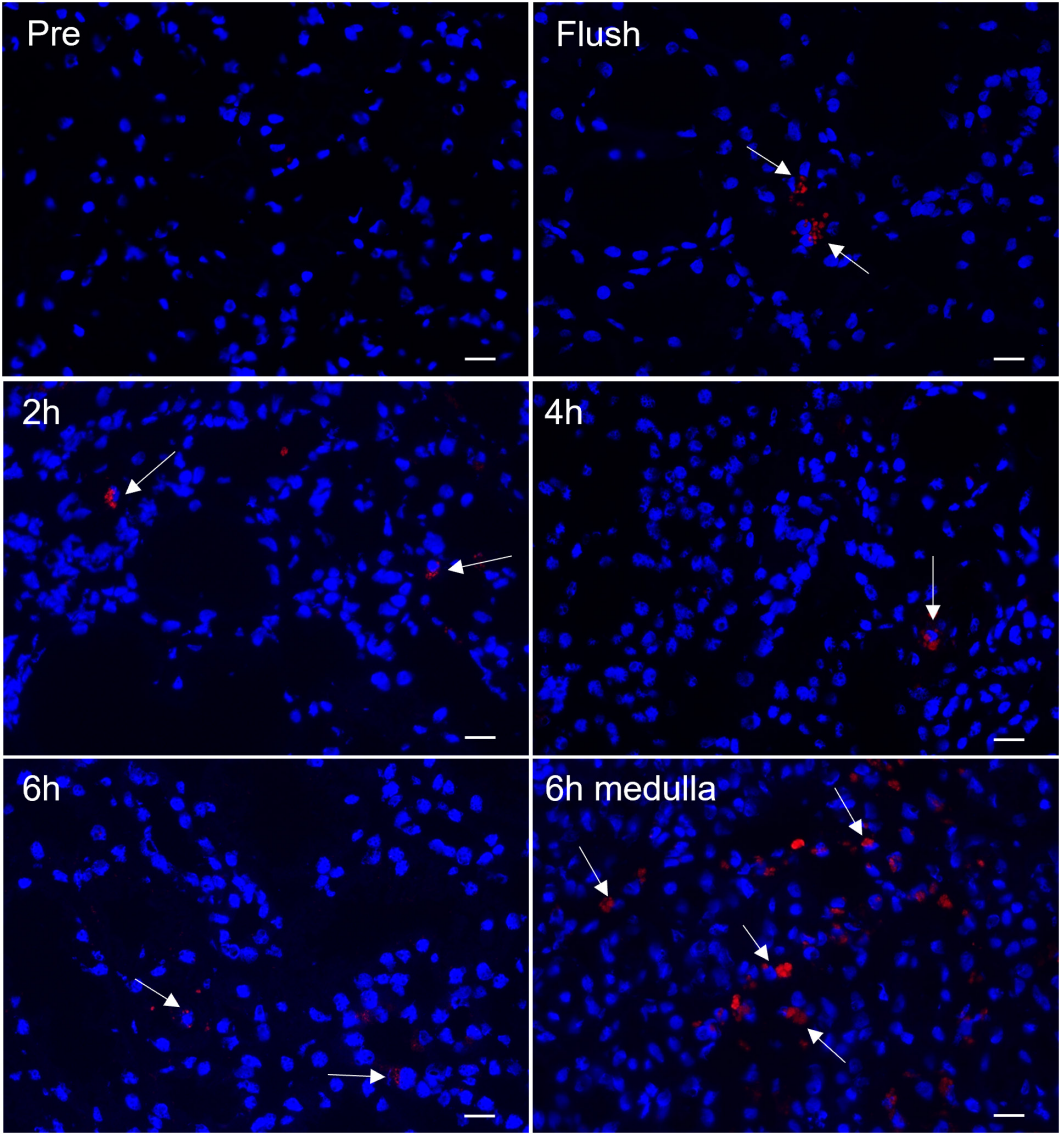
nKSPC can be traced within human kidneys perfused in normothermic machine perfusion (NMP). 10 million nKSPC were labeled with a red fluorescent dye (white arrows) and flushed into the kidneys before ex‐vivo NMP. Biopsies from the cortical region were collected before NMP (pre), immediately after nKSPC administration (flush), at 2, 4, and 6 h of perfusion, and from the medullary region at 6 h of NMP. Cryosections were counterstained only with the nuclear marker DAPI. Scale bar: 10 μm.

In order to follow the cells' fate during NMP, nKSPC were pre‐labeled with a red fluorescent dye. All cells injected into the renal artery were fluorescently red (Figure S1). Cryosections of biopsies were counterstained with DAPI for nuclear localization. We found nKSPC being present in the renal cortex immediately after infusion (flush), and at the later time points (2, 4, and 6 h) during NMP. By the end of perfusion, at 6 h, the majority of nKSPC were observed in the medullary region (Figure 2). Control kidneys had no red fluorescence (Figure S3).

### 3.3 nKSPC treatment induces de novo SIX2 expression in proximal tubule cells

#### 3.3.1 De novo SIX2 expression in human donor kidneys

**FIGURE 3:**
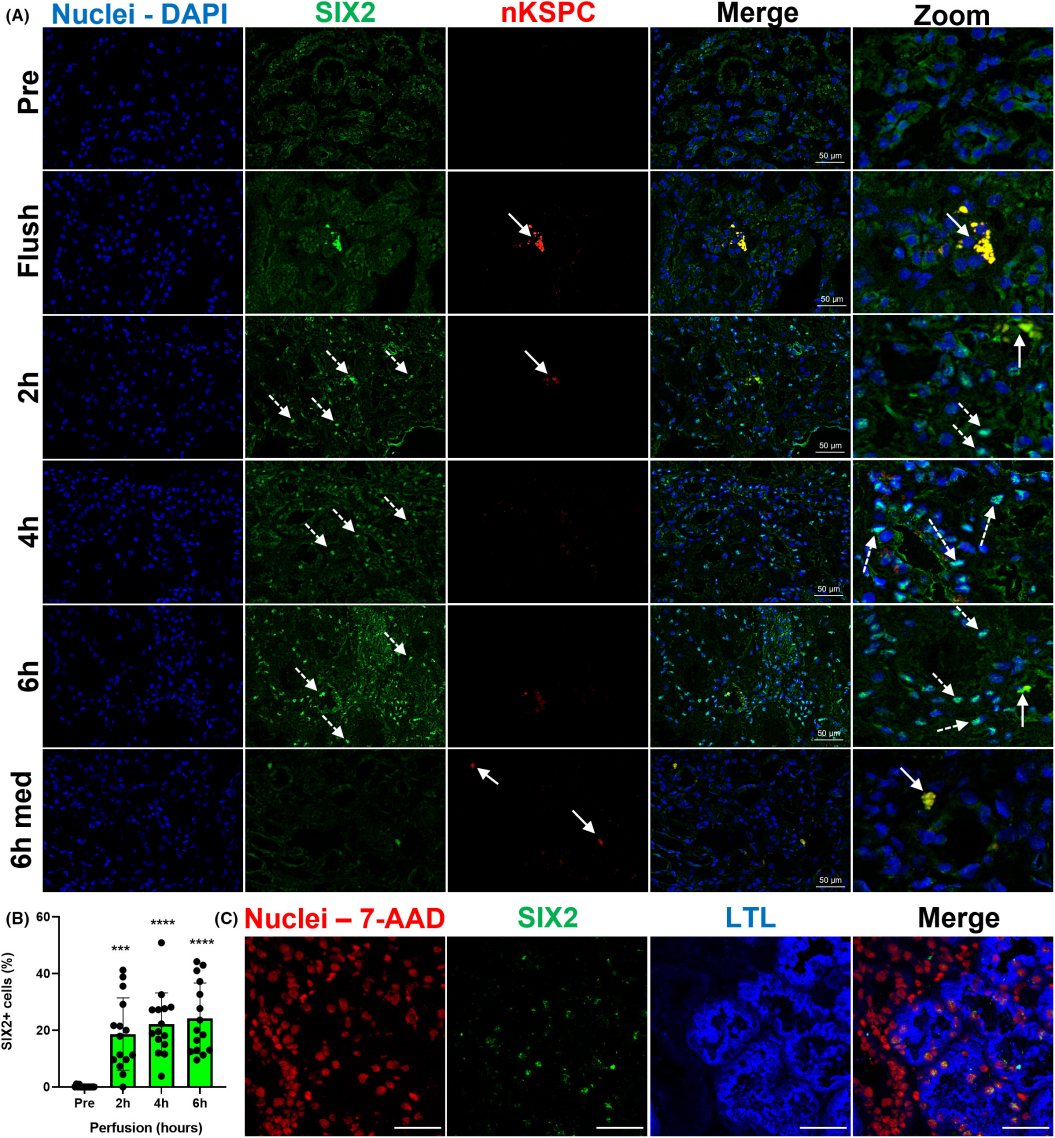
nKSPC administration induces de novo SIX2 activation in proximal tubules of injured human kidneys. (A) Representative images of SIX2 staining (green) in human kidney biopsies before (pre), after nKSPC administration (flush), and after 2, 4, and 6 h of normothermic machine perfusion (NMP) with labeled nKSPC (red). The specific nuclear staining of SIX2 was only observed at 2, 4, and 6 h of NMP at the cortical zone of the kidney. No signal was observed before infusion of nKSPC (pre) nor before starting NMP (flush) or at the medullary region (6 h med). Nuclei were counterstained with DAPI. Dashed arrows point at nuclear SIX2 staining and full arrows point at nKSPC. Scale bar: 50 μm. Zoomed images were added for better appreciation of details due to the known high background for SIX2 antibody. Note nKSPC (red) positive for SIX2 (green) in these merged images (yellow). (B) The graph represents the quantification of SIX2+ cells in human kidneys during NMP with nKSPC. For quantification, five images were analyzed per kidney (*n* = 3) per time point, in total 15 images per condition. Data were analyzed by one‐way ANOVA, with the Geisser–Greenhouse correction, comparing each time point with condition pre; ***p < .001, ****p < .0001. (C) Representative confocal images of SIX2 staining (green) and lectin (LTL ‐ blue) on human kidney biopsy after 6 h of normothermic machine perfusion (NMP) with nKSPC. The nuclei were counterstained with 7‐AAD (red), due to the combination of colors of secondary antibodies. Scale bar: 50 μm.

Because nKSPC express endogenous SIX2, we analyzed SIX2 expression in the kidney biopsies (Figure 3; Figure S4).

Before infusion of nKSPC (condition: pre), no SIX2 expression was observed. Immediately after infusion, SIX2+ nKSPC could be traced within the tissue. Strikingly, after 2 h of NMP + nKSPC we observed a de novo expression of SIX2 in the tubular cells of the donor kidneys, which increased at 4 h and peaked at 6 h in the cortex (Figure 3A,B). This phenomenon was unexpected, since to our knowledge SIX2 re‐expression in adult kidneys have never been reported, even upon injury.^7^ The SIX2+ cells were mainly co‐localized with LTL at the proximal tubules of the kidneys (Figure 3C), while the control did not show SIX2 expression (Figure S5).

#### 3.3.2 De novo SIX2 expression in human proximal tubule cells in vitro

**FIGURE 4:**
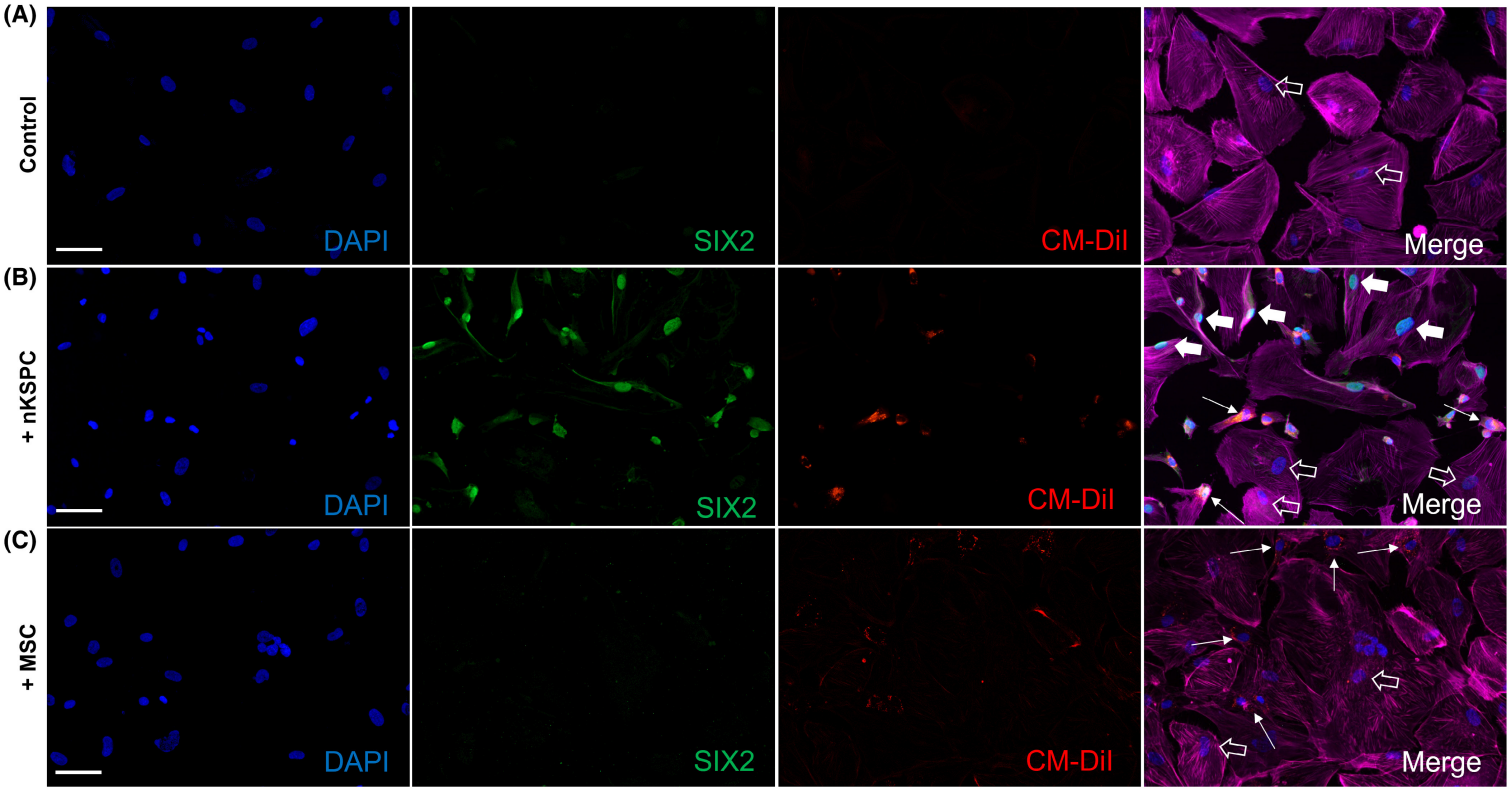
SIX2 staining of conditionally immortalized proximal tubule epithelial cells (PTEC) co‐cultured with neonatal kidney stem progenitor cells (nKSPC) or bone marrow mesenchymal stem cells (MSC). (A) Representative images of proximal tubule epithelial cells (PTEC) injured with 20 ng/ml of tumor necrosis factor‐α (TNF‐α) and 100 ng/ml of interferon‐gamma (IFN‐γ) in hypoxia (1% O_2_) in co‐culture with (B) CM‐DiI labeled nKSPC (red) or (C) MSC (red) for 24. Nuclei are stained with DAPI (blue), SIX2 is stained in green and Actin filaments (phalloidin) in magenta for better visualization of the whole cell surface. Arrows point at nKSPC or MSC, large full arrows point at SIX2 positive PTEC and empty arrows point at SIX2 negative PTEC. Note that only PTEC co‐cultured with nKSPC expressed SIX2. Scale bar: 100 μm.

To exclude confounder factors arising from the whole organ or the perfusion system, we developed an in vitro assay to model the PTEC‐nKSPC interactions. PTEC were exposed for 24 h to TNF‐α and IFN‐γ in hypoxia to mimic the ischemic environment of donor kidneys. These PTEC were then co‐cultured with nKSPC in a ratio of 1:1. Healthy or injured PTEC did not express SIX2, but incubation with nKSPC induced its expression similarly to the observations after administration of nKSPC in donor kidneys (Figure 4). To understand if this phenomenon was unique to nKSPC, the same assays were performed using MSC, which do not express SIX2. In fact, no SIX2 expression arose in injured PTEC upon co‐culture with MSC (Figure 4).

### 3.4 De novo SIX2 activation in human kidneys is accompanied by tubular cell proliferation and upregulation of regenerative markers

**FIGURE 5:**
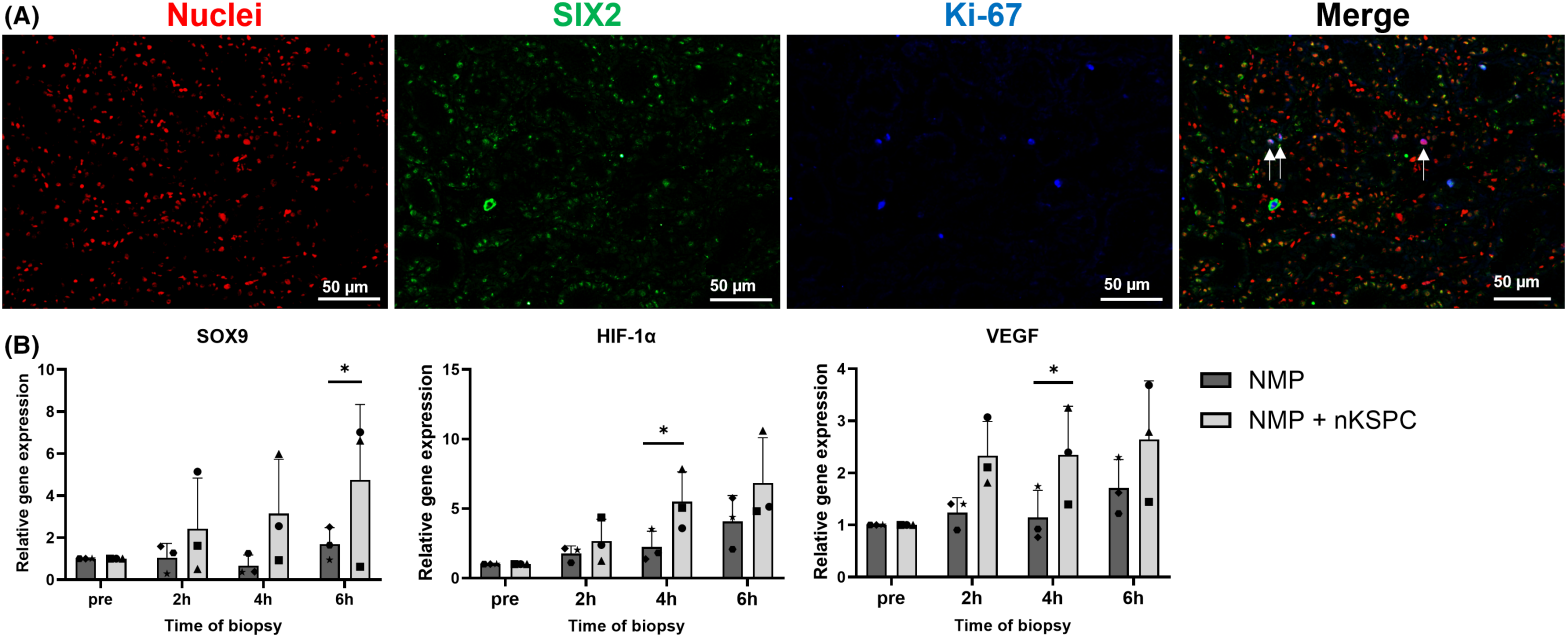
De novo SIX2 activation is accompanied by tubular cell proliferation and regeneration. (A) Representative images of double‐staining for SIX2 (green) and Ki67 (blue) on human cortex kidney biopsies after 6 h of normothermic machine perfusion (NMP) with nKSPC. Nuclei were counterstained with 7‐AAD and because it was possible that some of the red‐stained nuclei belonged to injected nKSPC, we only quantified the 6 h of NMP biopsies, since in the previous analysis, no or very rare nKSPC were found in the cortex at this time‐point. Arrows point at the double‐positive cells SIX2 + Ki67+. Scale bar: 50 μm. (B) Gene expression of hypoxia‐induced factor 1‐α (*HIF‐1α*) and vascular endothelial growth factor (*VEGF*) relative to β‐Actin in biopsies of human kidneys perfused with or without nKSPC in NMP. The individual kidneys are represented by a symbol and the respective demographic characteristics are described in Table 1. Results are expressed as mean ± SD of qPCR conducted in triplicate. Statistics were performed using multiple unpaired *t* tests, comparing NMP versus NMP + nKSPC per time point. * p < .05.

Ischemia activates a variety of intrinsic repair processes and the cell cycle to compensate for cell loss. We evaluated the proliferative state of the donor kidney cells by Ki67 staining. We found that very few cells were proliferating before NMP (condition: pre; Ki67+ cells = 0.53% ± 0.51), which doubled after 6 h (condition: NMP 6 h; Ki67+ cells = 1.29% ± 0.51 and NMP + nKSPC 6 h Ki67+ cells = 1.21% ± 0.61). Importantly, in NMP+ nKSPC, almost half of the proliferating cells were also SIX2+ (condition: NMP + nKSPC 6 h; Ki67 + SIX2+ cells = 40.6% ± 10.8), while in NMP control, none of the Ki67+ cells expressed SIX2 (Figure 5A).

It has been suggested that Sox9+ cells regenerate proximal tubule epithelium after renal injury in mice.^14^ We analyzed *SOX9* expression in our samples and observed a significant increase in the NMP + nKSPC condition in comparison with NMP control kidneys (Figure 5B). In addition, activation of hypoxia‐inducible factors (HIFs) plays an important role in kidney injury and repair by regulating HIF target genes,^15^ such as upregulation of vascular endothelial growth factor (VEGF).^16^, ^17^ We measured the expression of *HIF‐1α* and *VEGF* in the perfused kidneys and found that both factors were significantly upregulated after 4 h of NMP + nKSPC (Figure 5B). These results corroborate with the concept of an ongoing effective regenerative process induced by NMP + nKSPC.

### 3.5 nKSPC administration has immunomodulatory effects in human kidneys

**FIGURE 6:**
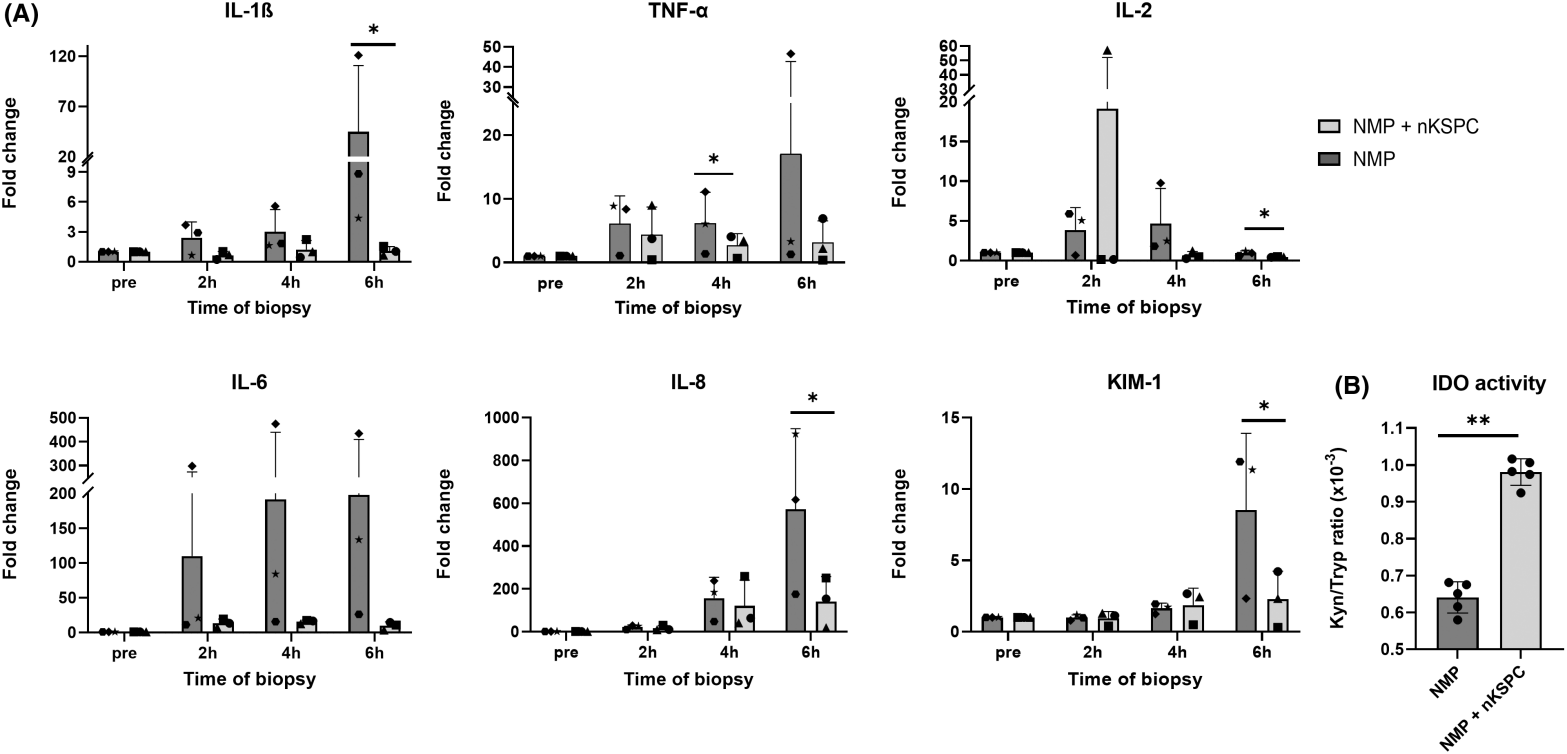
Evaluation of the immunomodulatory potential of nKSPC treatment during NMP. (A) Gene expression (qPCR) analysis of cytokines in biopsies of human kidneys perfused with or without nKSPC. Results are relative to β‐Actin and normalized to the expression before perfusion (pre). Results are expressed as mean ± SD of qPCR conducted in triplicate. Statistical analysis was performed using multiple unpaired *t* tests, comparing NMP versus NMP + nKSPC per time point. * p < .05. The individual kidneys are represented by a symbol and the respective demographic characteristics are described in Table 1. (B) Kynurenine/Tryptophan ratio representing IDO activity in samples of the perfusate during NMP and NMP + nKSPC from 2 to 6 h, at every hour. Each perfusate sample was measured in triplicate. Dots represent the mean of measurement of the three kidneys every hour of NMP (2; 3; 4; 5 and 6 h) and the bars represent the mean ± SD of all measurements; p < .01.

MSCs are known to induce immunomodulation in solid organ transplantation.^18^, ^19^, ^20^ Likewise, we evaluated the immunomodulatory potential of nKSPC in human kidneys during NMP. nKSPC treatment significantly reduced the expression of the pro‐inflammatory cytokines IL‐1β, TNF‐α, IL‐2, and IL‐8 in comparison with the control kidneys (Figure 6A). There was a numerical reduction of the pro‐inflammatory cytokine IL‐6 in NMP + nKSPC but this did not reach statistical significance (𝑝 = .092). Importantly, the biomarker of kidney injury KIM‐1 was significantly reduced in NMP + nKSPC in comparison with NMP control (Figure 6A).

**FIGURE 7:**
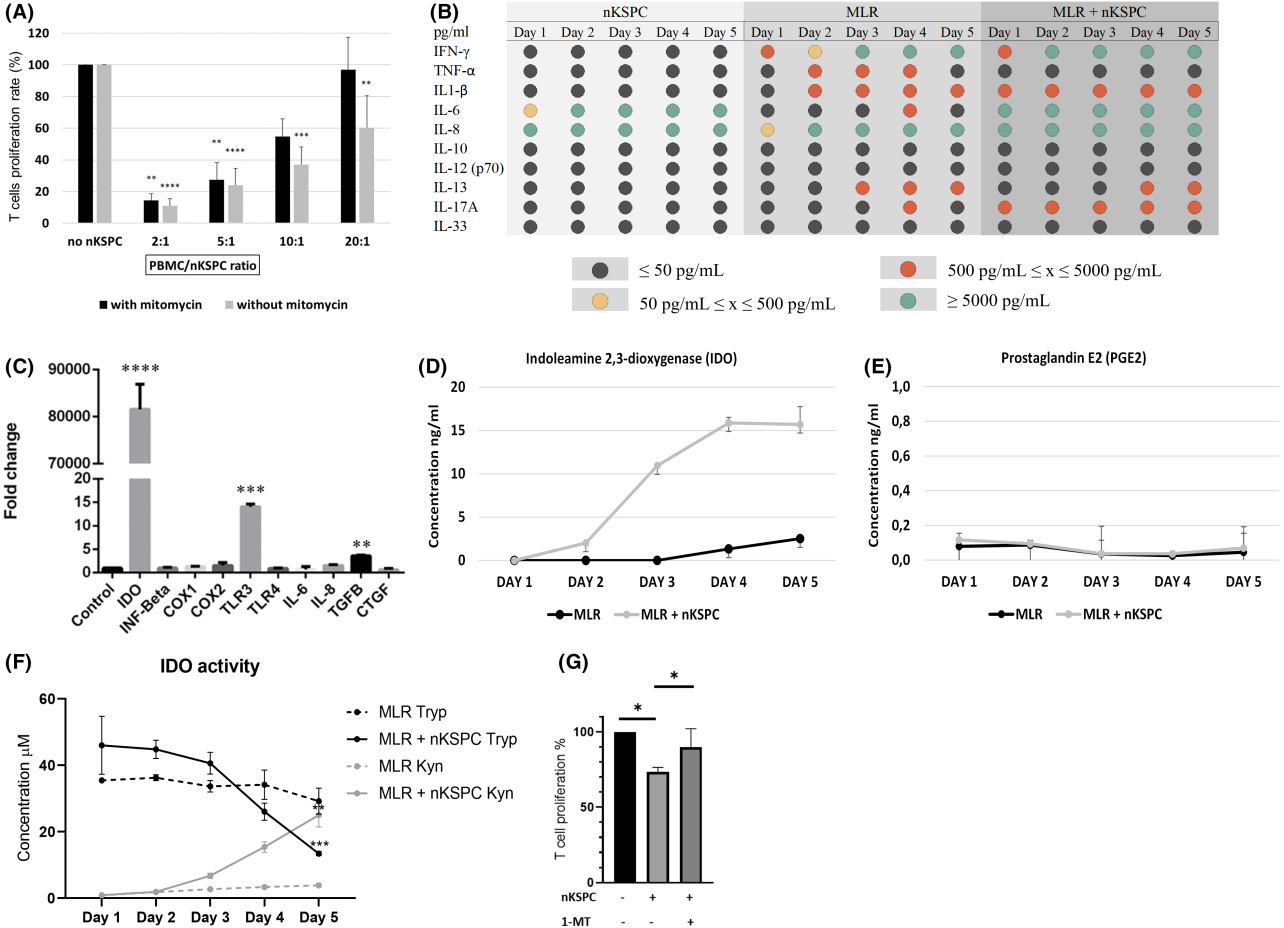
nKSPC inhibit allogeneic T cell responses by tryptophan degradation via the indoleamine 2,3‐dioxygenase–mediated (IDO) pathway. (A) Peripheral blood mononuclear cells (PBMC) were stimulated with RPMI 1788 cells, in the absence or the presence of nKSPC at different ratios (PBMC/nKSPC: 0, 2:1, 5:1, 10:1, and 20:1). Nkspc were treated with mitomycin C to hinder proliferation during co‐culture and to ensure the known number of cells. The proliferation rate was quantified after 5 days of co‐culture. Data are expressed as mean ± SD of 3 independent experiments conducted in triplicate. 100% of proliferation corresponds to PBMC stimulated with RPMI 1788 cells only. Data were analyzed by one‐way ANOVA test: ** p < .01, *** p < .001, **** p < .0001 PBMC stimulated with RPMI 1788 without nKSPC versus PBMC stimulated with RPMI 1788 in co‐culture with nKSPC. (B) A multiplex human cytokine detection (MSD U‐plex assay) was used to measure levels of secretory factors in cell culture supernatants for 5 consecutive days from nKSPC alone and MLR with or without nKSPC. Black dot, value ≤50 pg/ml; yellow dot, 50 pg/ml ≤ value ≤500 pg/ml; red dot, 500 pg/ml ≤ value ≤5000 pg/ml; green dot, value ≥5000 pg/ml. (C) mRNA expression levels of various immunomodulatory genes in nKSPC primed with 200 IU/ml of IFN‐γ for 24 h and normalized to baseline nKSPC (control). Data are expressed as relative quantification to β‐Actin and normalized to nKSPC control using the ΔΔCt method. Results show mean ± SD of 3 independent experiments conducted in triplicate. Data were analyzed by Student's 𝑡‐test (paired, 2‐tailed); ** p < .01, *** p < .001, **** p < .0001. Genes: Indoleamine 2,3‐dioxygenase (*IDO*), Interferon‐beta (*IFN‐beta*), cyclo‐oxygenase‐1 (*COX‐1*), cyclo‐oxygenase‐2 (*COX‐2*), toll‐like receptor‐3 (*TLR‐3*), toll‐like receptor‐4 (*TLR‐4*), interleukin‐6 (*IL‐6*), interleukin‐8 (*IL‐8*), transforming growth factor‐beta (*TGFB*) and connective tissue growth factor (*CTGF*). (D, E) Quantification of soluble human IDO and prostaglandin E2 (PGE2) were measured in the supernatant of MLR co‐cultures for 5 days without (MLR) or with neonatal kidney stem/progenitor cells (MLR + nKSPC) in a 2:1 PBMC/nKSPC ratio. (F) Tryptophan (Tryp) and kynurenine (Kyn) were measured by HPLC‐MS in the supernatant of MLR and MLR + nKSPC during 5 days of co‐culture. Results are expressed as mean ± SD of three independent experiments conducted in triplicate. Statistics were performed using multiple unpaired *t* tests, comparing MLR versus MLR + nKSPC per time point. Statistical significance was determined using the Holm‐Sidak method, with α = .05. (G) Effect of the IDO inhibitor 1‐methyl tryptophan (1‐MT) in MLR co‐culture with nKSPC for 3 days. Results are expressed as mean ± SD, data were analyzed by one‐way ANOVA test: * p = .01.

One of the most accepted mechanisms of immunomodulation of MSC is the upregulation of IDO.^21^ IDO catalyzes the tryptophan degradation along the kynurenine pathway. Tryptophan depletion in the microenvironment and accumulation of kynurenine, inhibits the activation, proliferation, and functional activity of T cells.^22^ We found a significant increase in IDO activity in kidneys treated with NMP + nKSPC in comparison with controls, which was demonstrated by the elevated kynurenine/tryptophan ratio (Figure 6B). This immunomodulatory mechanism of nKSPC was also confirmed in vitro (Figure 7).

### 3.6 nKSPC inhibit allogeneic T cell responses by tryptophan degradation via the IDO pathway

The immunomodulatory effect seen in nKSPC‐treated kidneys is of particular interest in the context of kidney transplantation. As alloreactive lymphocytes are the primary mediators of kidney graft rejection, we assessed whether nKSPC could inhibit allogenic T cell responses when co‐cultured in MLR. We detected that nKSPC hinder T cell proliferation in a dose‐dependent manner (Figure 7A). A ratio of at least 5:1 PBMC/nKSPC (1 × 10^5^: 2 × 10^4^) was necessary to significantly halt T cell proliferation. As the maximum suppressive effect was desired, in the mechanistic studies we used the ratio 2:1 PBMC/nKSPC (1 × 10^5^: 5 × 10^4^), which decreased T cell proliferation to 14.4% ± 6.0 of the initial response in the absence of nKSPC.

Next, we analyzed the cytokines and chemokines secreted by nKSPC alone as well as in the MLR with and without the addition of nKSPC. We found that nKSPC at baseline secreted high levels of IL‐6 and IL‐8 (≥5000 pg/ml) (Figure 7B). In the MLR without nKSPC high concentrations of INF‐γ, TNFα, IL‐1β, IL‐6, IL‐8, IL‐13, and IL‐17A were measured. Importantly, co‐culture with nKSPC decreased the release of the pro‐inflammatory cytokine TNF‐α, but increased IFN‐γ and IL‐17A, which are known to mediate the immunosuppressive capacity of MSC.^22^, ^23^ Therefore, we primed nKSPC with IFN‐γ and analyzed the differential gene expression. As shown in Figure 7C, the expression of IDO was highly upregulated (qPCR Ct value from 36 to 21) in comparison with other genes. So, we measured the concentration of soluble IDO released in the supernatant of the MLR, in which cytokines were measured. We observed a steep increase of IDO in MLR + nKSPC, reaching saturation on day 4 (Figure 7D).

As other mediators affected by IFN‐γ could be involved in the immunomodulatory mechanism, we also measured the concentration of prostaglandin E2 (PGE2). However, almost no PGE2 could be detected in the secretome of MLR + nKSPC (Figure 7E), which correlated with no significant upregulation of COX‐1 and COX‐2^24^ genes when nKSPC were primed with IFN‐γ (Figure 7C). These results strongly suggest that IDO‐driven tryptophan degradation is the major mechanism of nKSPC immunomodulation. Hence, we measured the concentration of tryptophan and kynurenine in the MLR supernatants. MLRs without nKSPC had high levels of tryptophan and very low levels of kynurenine throughout the period of 5 days (Figure 7F—dashed lines). MLR + nKSPC showed a significant reduction of tryptophan starting from day 3 whereas kynurenine, the tryptophan metabolite, significantly increased (Figure 7F—full lines). Moreover, adding IDO‐inhibitor to the MLR + nKSPC showed a restoration of T cell proliferation (Figure 7G).

## 4 DISCUSSION

This study was designed to provide proof of principle evidence that nKSPC can be safely administered to human donor kidneys during NMP. We found that nKSPC treatment induced the re‐activation of SIX2 expression in proximal tubular epithelial cells of the donor kidneys upon perfusion. The de novo activation of factors specifically and uniquely responsible for nephron formation, such as *SIX2*, had so far never been reported.^7^, ^25^ This phenomenon was followed by upregulation of other regenerative markers such as SOX9 and VEGF, which suggests the de novo expression of SIX2 as the initiation of an endogenous regenerative process.

In our study, the majority of Ki67+ donor cells were also SIX2+, demonstrating the active proliferation of the cells expressing SIX2. Higher levels of *SOX9* and *VEGF* expression might be induced by the upregulation of *HIF‐1α* ^*14*^. Studies demonstrated that *HIF* activation decreases renal cell apoptosis in AKI and upregulation of *HIF‐1α* promotes kidney tissue repair, being critical for proximal tubule cell survival and facilitating cell proliferation.^15^, ^26^ VEGF has been implicated as a critical factor for renal recovery after IRI due to its mitogenic, angiogenic, anti‐inflammatory, and antiapoptotic effect.^16^

Based on the analysis of these tissue regeneration markers (HIF‐1α, VEGF, and SOX9) we can assume the regenerative superiority of the treatment with nKSPC compared to NMP only.

DCD kidneys have a high incidence of acute tubular necrosis and subsequent delayed graft function. Therefore, promoting epithelial cell regeneration is a great advantage for successful graft quality improvement and survival. A few other studies support the potential of cell therapy during kidney perfusion using other human cell types,^20^, ^27^ but so far, only one study demonstrated a possible epithelial cell regeneration characterized by increased ATP synthesis, normalization of the cytoskeleton, and increased mitosis, followed by significantly increased production of growth factors that could be associated with regenerative pathways after ischaemic insults.^27^

Although our developed in vitro co‐culture model indicated that MSCs do not activate SIX2 in PTECs, the infusion of other cell types during NMP would be an important control to understand the possible advantage of nKSPC treatment. In addition, this study was limited to elucidate the mechanistic pathways leading to SIX2 reactivation in donor kidney cells. Longer periods of perfusion will be required to elucidate the consequences of SIX2 activation.

Tracking labeled nKSPC in the perfused kidneys showed that nKSPC were clustered immediately after infusion (condition: flush), with single cells or fragments found at 2 h and 4 h, while at 6 h nKSPC signal could barely be observed in the cortex of the kidneys, but a high number of cells/fragments was observed in the medullary region (Figure 2). The latter observation was also demonstrated when multipotent adult progenitor cells (MAPC) were perfused in human kidneys for 7 h and were found in high amounts in the medullary region.^20^ The attraction of nKSPC to the renal medulla might be due to the fact that tubules of the S3 segment region are the most sensitive regions to IRI.^28^ Not by coincidence, in another study, MSC treatment showed a positive effect on ATP synthesis, which was more pronounced in the medullary region, suggesting increased synthetic functions associated with regeneration of damaged tubules.^27^

Whilst perfusion of donor kidneys is an excellent pre‐clinical model to test novel treatments with translational potential, they do have high biological variability. This study is limited to a small sample size and a heterogenous group of DCD kidneys declined for transplantation due to damage in three cases, poor in‐situ perfusion in two, and a diseased artery in one. All donors had a normal renal function and the kidneys were of good quality at the time of organ retrieval but were subject to prolonged cold ischemic injury. All kidneys functioned well during NMP and produced urine. The level of RBF was similar between groups suggesting that the nKSPC had no adverse effects. Although one donor was younger and CIT was shorter than in other kidneys (NMP + nKSPC #3), the cytokine measurements and effects of nKSPC treatment to induce SIX2 expression were similar to the other kidneys of the same group.

Kidneys treated with nKSPC had a reduction of inflammatory cytokines and biomarkers of kidney injury. There was a trend toward an increase in the volume of urine produced and lower urinary NGAL. These parameters have been used for the functional assessment of kidneys in NMP and, after transplantation, it was shown that they correlated with improved clinical outcomes.^4^ Better performance of these key parameters has also been suggested as a sign of less tissue damage, better cellular metabolism, improved tissue perfusion, and re‐establishment of homeostasis.^20^ Moreover, NMP + nKSPC administration resulted in lower levels of KIM‐1, a transmembrane tubular protein, which is undetectable in normal kidneys but is markedly induced after injury and its high excretion in urine predicts long‐term renal graft loss.^29^

In conclusion, the administration of nKSPC in NMP of injured human kidneys is safe and feasible. This proof of principle study demonstrated that nKSPC can potentiate immunomodulation and trigger endogenous regeneration. Therefore, nKSPC may serve as a potent source of cells for kidney‐targeted cell therapy aiming at the improvement of kidney graft quality ultimately attenuating the problem of shortage of organs available for transplantation.

### AUTHOR CONTRIBUTIONS

Conceptualization: FOA, SH, LvdH, EL; data curation: FOA, SH; formal analysis: FOA, SH, JH; investigation: FOA, SH, NJ, TE, JH, KV, SA; methodology: FOA, JH, SH, LvdH, MN, EL; project administration: FOA; resources: FOA, JH, SH, LvdH, KV, MN, EL; supervision: LvdH, MN, EL; validation: FOA, SH; visualization: FOA, SH, KV; funding acquisition: FOA, EL; writing—original draft preparation: FOA, SH; writing—review and editing: FOA, SH, KV, SA, KV, JH, NJ, TE, MN, LvdH, EL.

## DISCLOSURE

The authors of this manuscript have no conflicts of interest to disclose as described by the *American Journal of Transplantation*.

## Supporting information


Appendix S1

## Data Availability

The data that support the findings of this study are available in the supplementary material of this article, additional information is available from the corresponding author upon reasonable request.
