# Supplementary material for: De novo SIX2 activation in human kidneys treated with neonatal kidney stem/progenitor cells
Source: Am J Transplant. 2022 Aug 11;22(12):2791–803. doi: 10.1111/ajt.17164 (PMC10087644; doi:10.1111/ajt.17164)

**SUPPLEMENTAL MATERIAL**

**TABLE OF CONTENTS**

**Supplementary Table 1:** Primers used to test mRNA level via qPCR.

**Supplementary Table 2:** Antibodies used in immunofluorescent staining.

**Supplementary Figure 1:** Validation of SIX2 antibodies and optimization of staining with CM-DiI for cells tracking.

**Supplementary Figure 2:** Mixed lymphocyte reaction co-culture with bone marrow derived mesenchymal stem cells.

**Supplementary Figure 3:** Immunofluorescent images of control human kidneys in NMP.

**Supplementary Figure 4:** RT-qPCR analysis of SIX2 gene expression in kidney biopsies and control samples.

**Supplementary Figure 5:** SIX2 staining of control human kidneys.

| **Target gene** | **GeneID** | **Protein** | **Forward primer (5’-3’)** | **Reverse primer (5’-3’)** |
| --- | --- | --- | --- | --- |
| *ACTB* | 60 | β-Actin | AAGAGCTACGAGCTGCCTGA | GACTCCATGCCCAGGAAGG |
| *SIX2 (qPCR)* | 10736 | SIX homeobox 2 | GCCGAGGCCAAGGAAAGGGAGAA | AGCAGTGCGGGGCTGGATGA |
| *IL-1β* | 3553 | Interleukin 1*β* | ATTACAGTGGCAATGAGGATGA | TGTAGTGGTGGTCGGAGAT |
| *IDO* | 3620 | Indoleamine 2,3-dioxygenase 1 | GCGCTGTTGGAAATAGCTTC | CAGGACGTCAAAGCACTGAA |
| *PTGS1/COX1* | 5742 | Prostaglandin-endoperoxide synthase 1 | CTTGGGCCATGGGGTAGAC | TCTACCGAGGGCGGGTACA |
| *PTGS2/COX2* | 5734 | Prostaglandin-endoperoxide synthase 2 | CTGGCAGGGTTGCTGGTG | CTTCAGCATAAAGCGTTTGCG |
| *TLR3* | 7098 | Toll like receptor 3 | AGCCTTCAACGACTGATGCT | TTTCCAGAGCCGTGCTAAGT |
| *TLR4* | 7099 | Toll like receptor 4 | CCTGTGCAATTTGACCATTG | AAGCATTCCCACCTTTGTTG |
| *TGFβ* | 7040 | Transforming growth factor beta 1 | CACATCAGAGCTCCGAGAAGCG | TAGTGAACCCGTTGATGTCCACT |
| *CCN2/ CTGF* | 1490 | Cellular communication network factor 2 | TCGGCTCCCCGGCCAACCGCAAGAT | CTCTGTGGTCTGGACCAGGCAGTT |
| *IL-2* | 3558 | Interleukin 2 | GGGATCTGAAACAACATTCATGTG | AGTCAGTGTTGAGATGATGCTTTG |
| *IL-6* | 3569 | Interleukin 6 | TACCCCCAGGAGAAGATTCC | GCCATCTTTGGAAGGTTCAG |
| *CXCL8 (IL-8)* | 3576 | Interleukin 8 | CTCTGCACCCAGTTTTCCTT | CTCTGCACCCAGTTTTCCTT |
| *HAVCR1* | 26762 | Kidney injury molecule 1 | ATCCTACATCTGGCAGATTCT | CTTATAGCGTGTGTCCTT |
| *HIF-1α* | 3091 | Hypoxia inducible factor 1 subunit alpha | TGACCAGCAACTTGAGGAAG | CCATCGGAAGGACTAGGTGT |
| *VEGFA*  *TNF-α* | 7422  7124 | Vascular endothelial growth factor A  Tumor Necrosis Factor alpha | ATGAACTTTCTGCTGTCTTGGGTGC  CACGCTCTTCTGCCTGCTGCACTTT | TGATTCTGCCCTCCTCCTTCTGC  TCAGCTTGAGGGTTTGCTACAAC |

**Supplementary Table 1.** Sequence of primers used for qPCR.

**Supplementary Table 2:** Antibodies used in immunofluorescent stainings.

|  | **Name** | **Target** | | **Manufacturer** | **Catalog #** | **Dilution** |
| --- | --- | --- | --- | --- | --- | --- |
| *Primary antibodies* | | | | | | |
|  | mouse anti-SIX2  rabbit anti-SIX2 | | SIX2  SIX2 | Abnova  Proteintech | H00010736-M01  11562-1-AP | 1:500  1:400 |
|  | biotin-conjugated Lotus tetragonolobus lectin | | LTL | Vector Laboratories | B-1325-2 | 1:50 |
|  | biotin-conjugated Ki-67  Alexa Fluor™ Plus 647 Phalloidin | | Ki67  Actin | Thermo Fisher  Invitrogen™ | 13-5698-82  A30107 | 1:50  1:400 |
| *Secondary antibodies* | | | | | | |
|  | donkey anti-mouse IgG H&L Alexa Fluor^®^ plus 488 | |  | Abcam | ab150105 | 1:400 |
|  | Streptavidin Alexa Fluor™ 405 | |  | Thermo Fisher | S32351 | 1:100 |

**Supplementary Figure 1: A)** nKSPC and bone marrow MSC labelled with the red dye CM-DiI after 48h incubation. 100% of the cells retained the red fluorescent dye for at least 48h. Slides were counterstained only with the nuclear marker DAPI (blue). Magnification: 200x; scale bar: 100µm. **B)** SIX2 staining in nKSPC using two different antibodies: SIX2 a is a mouse derived monoclonal antibody from Abnova (H00010736-M01). Six2 b is a rabbit derived polyclonal antibody from Proteintech (11562-1-AP). nKSPC were stained for SIX2 (green) according to the protocol described in material and methods and images were analysed with the same magnification and light exposure time. Magnification: 200x; scale bar: 100µm.


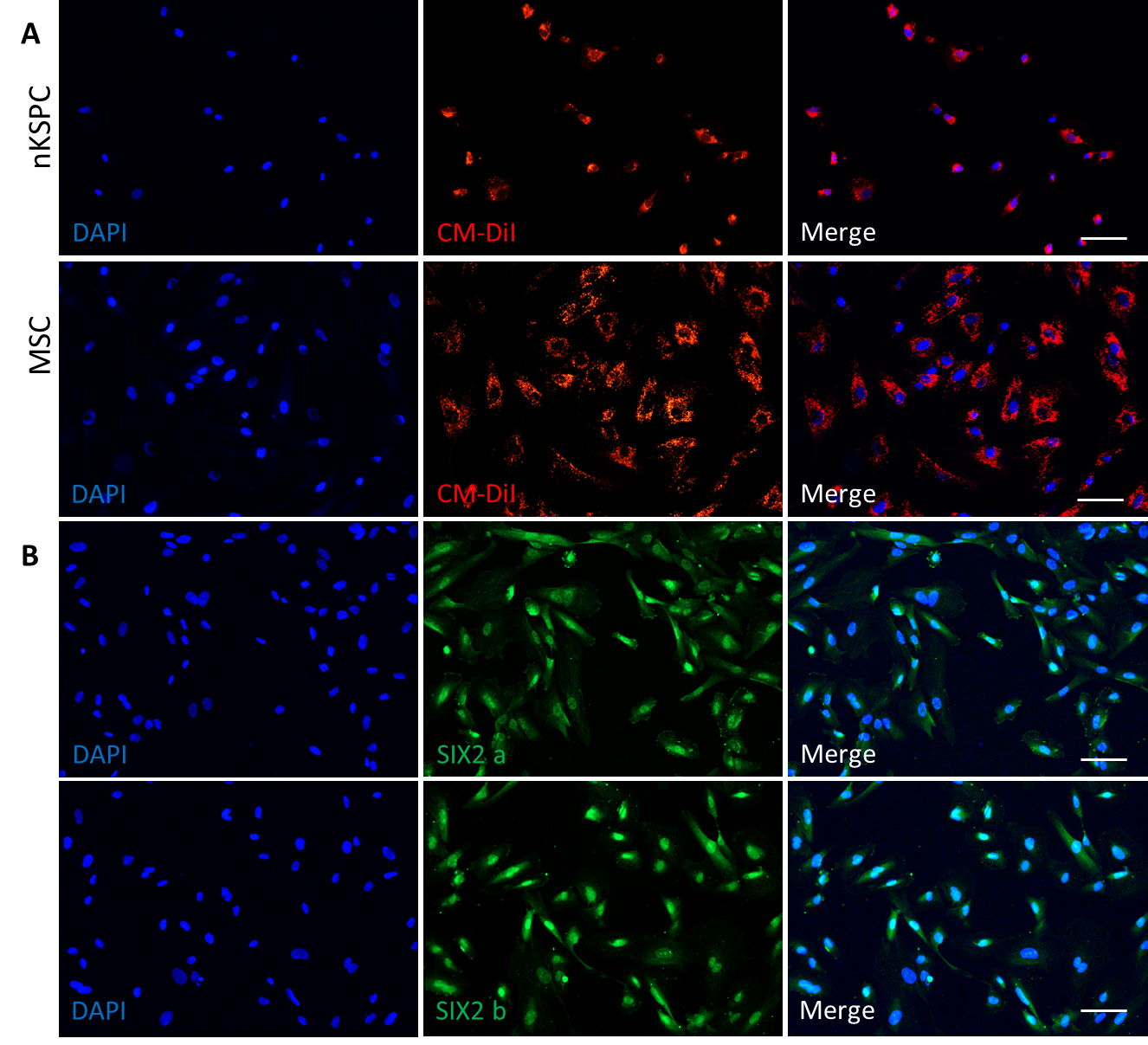


**Supplementary Figure 2:** Mixed lymphocyte reaction co-culture with bone marrow derived mesenchymal stem cells (MSC). Peripheral blood mononuclear cells (PBMC) were stimulated with RPMI 1788 cells, in the absence or the presence of MSC at different ratios (PBMC / MSC: 0, 2:1, 5:1, 10:1 and 20:1). MSC were treated with mitomycin C to hinder proliferation during co-culture and to ensure the known number of cells. The proliferation rate was quantified after 5 days of co-culture. Data are expressed as mean ± SD of 3 independent experiments conducted in triplicate. 100% of proliferation corresponds to PBMC stimulated with RPMI 1788 cells only. Data were analysed by one-way ANOVA test: ** 𝑝 < 0.01, *** 𝑝 < 0.001, **** 𝑝 < 0.0001 PBMC stimulated with RPMI 1788 without MSC versus PBMC stimulated with RPMI 1788 in co-culture with MSC.


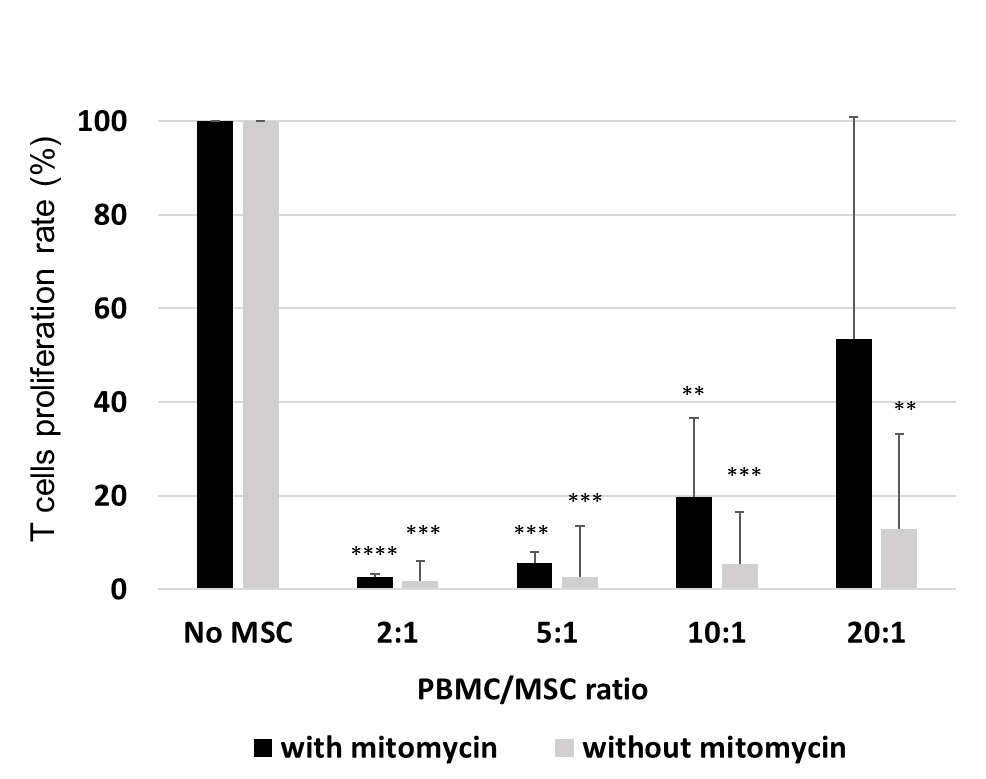


**Supplementary Figure 3:** Immunofluorescent images of human kidneys in NMP without nKSPC administration. Cryosections were counterstained only with the nuclear marker DAPI. Biopsies were taken from the kidney cortex before (pre), at 2, 4 and 6 hours of NMP and from the medullary region at 6h of NMP. Scale bar: 10µm.


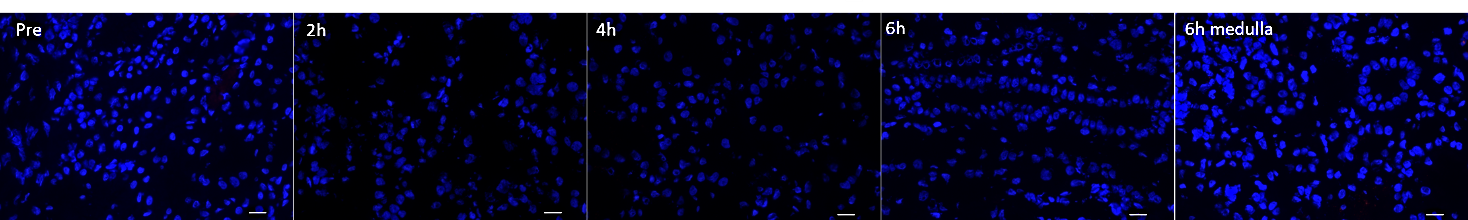


**Supplementary Figure 4:** Product of qRT-PCR analysis of *SIX2* expression. SIX2 expression in 1) nKSPC, 2) biopsies of human kidney cortex before NMP (pre and flush), at 2, 4 and 6 hours of NMP and from the medullary region at 6h of NMP + nKSPC administration and 3) control foetal kidney of 19 weeks gestational age and commercial adult kidney cDNA. Product length *SIX2*: 152bp; *β-actin*: 101bp.


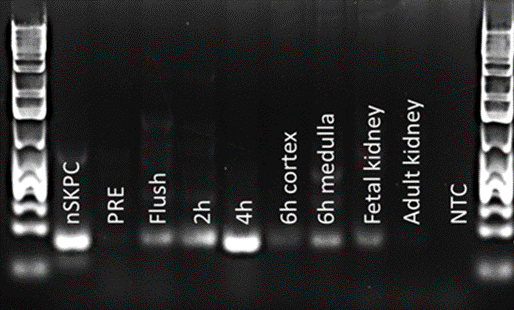


*SIX2*

**200 bp**

**75 bp**

**75 bp**

**200 bp**


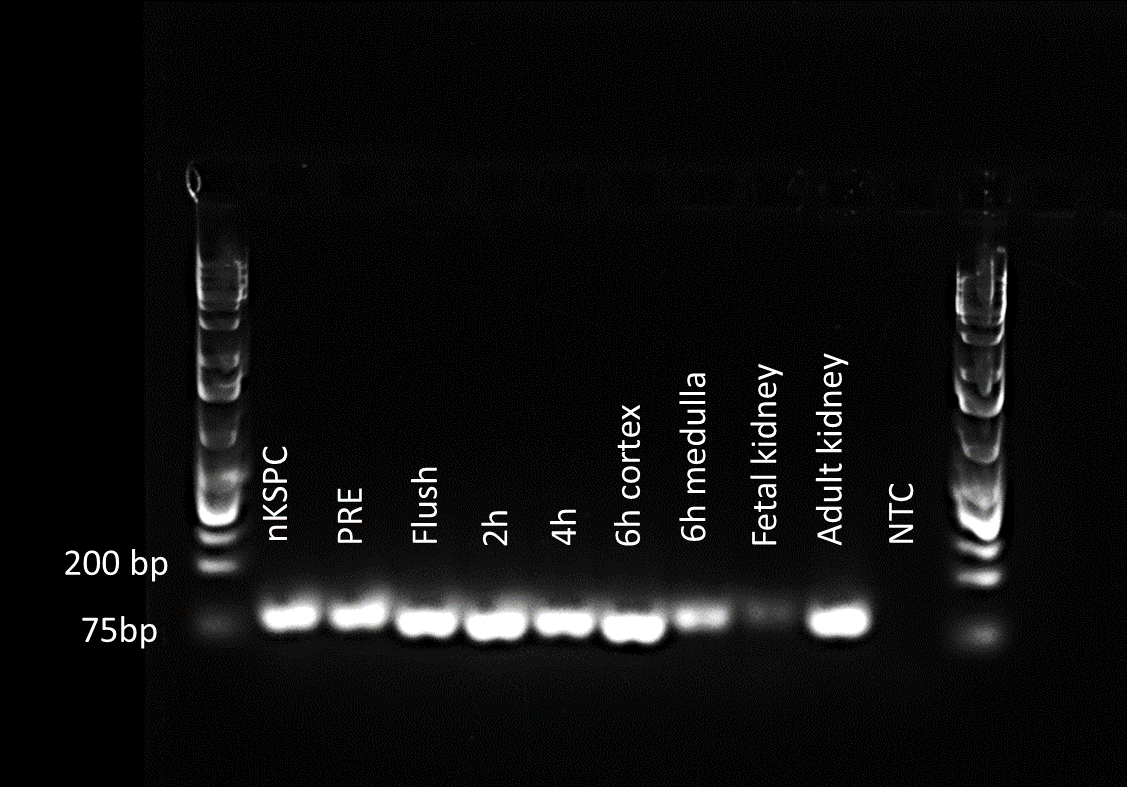


*β-actin*

**Supplementary Figure 5:** SIX2 staining of control human kidneys. Representative images of SIX2 staining (green) in human cortex kidney biopsies before (pre) and after 2, 4 and 6 hours of normothermic machine perfusion (NMP) without nKSPCs. Nuclei were counterstained with DAPI. Scale bar: 50 µm.


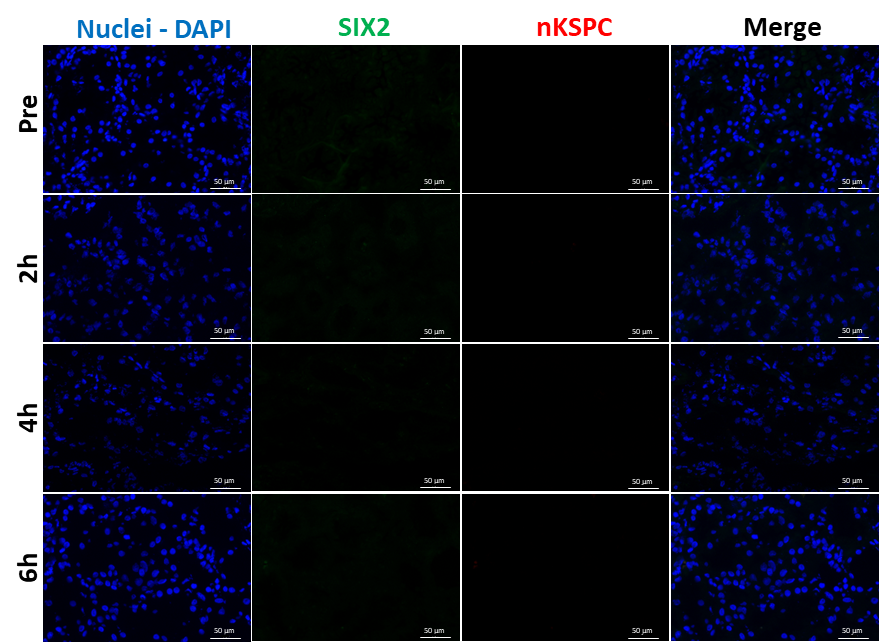

Supplement: Supplementary file 1 — Appendix S1 [file AJT-22-2791-s001.docx]
